# Verification of quality parameters for portal images in radiotherapy

**DOI:** 10.2478/v10019-010-0052-6

**Published:** 2010-12-31

**Authors:** Csilla Pesznyák, István Polgár, Csaba Weisz, Réka Király, Pál Zaránd

**Affiliations:** 1 Uzsoki Hospital, Municipal Centre for Oncoradiology, Budapest, Hungary; 2 Budapest University of Technology and Economics, Institute of Nuclear Techniques, Budapest, Hungary

**Keywords:** electronic portal imaging device, quality control, portal film

## Abstract

**Background:**

The purpose of the study was to verify different values of quality parameters of portal images in radiotherapy.

**Materials and methods:**

We investigated image qualities of different field verification systems. Four EPIDs (Siemens OptiVue500aSi^®^, Siemens BeamView Plus^®^, Elekta iView^®^ and Varian PortalVision™) were investigated with the PTW EPID QC PHANTOM^®^ and compared with two portal film systems (Kodak X-OMAT^®^ cassette with Kodak X-OMAT V^®^ film and Kodak EC-L Lightweight^®^ cassette with Kodak Portal Localisation ReadyPack^®^ film).

**Results:**

A comparison of the f50 and f25 values of the modulation transfer functions (MTFs) belonging to each of the systems revealed that the amorphous silicon EPIDs provided a slightly better high contrast resolution than the Kodak Portal Localisation ReadyPack^®^ film with the EC-L Lightweight^®^ cassette. The Kodak X-OMAT V^®^ film gave a poor low contrast resolution: from the existing 27 holes only 9 were detectable.

**Conclusions:**

On the base of physical characteristics, measured in this work, the authors suggest the use of amorphous-silicon EPIDs producing the best image quality. Parameters of the EPIDs with scanning liquid ionisation chamber (SLIC) were very stable. The disadvantage of older versions of EPIDs like SLIC and VEPID is a poor DICOM implementation, and the modulation transfer function (MTF) values (f50 and f25) are less than that of aSi detectors.

## Introduction

Electronic Portal Imaging Devices (EPIDs) are used for patient setup during radiotherapy sessions.^1^–^6^ At the same time amorphous silicon (aSi) detectors also offer the possibility of implementing transit dosimetry – this, however, requires a very good quality control protocol.^7^–^10^ A good quality control process comprises a series of procedures to be carried out regularly, with the aim of which the user may ascertain that the equipment provides good image quality and correct measured data. Users usually realize only a sudden drastic worsening of the image quality and fail to notice gradual worsening.

We tested the image qualities of different field verification systems. Four different EPIDs (Siemens OptiVue500aSi^®^, Siemens BeamView Plus^®^, Elekta iView^®^ and Varian PortalVision™) and two Kodak films (the X-OMAT V^®^ film in a X-OMAT^®^ cassette and the Portal Localisation ReadyPack^®^ film in a EC-L Lightweight^®^ cassette) were examined with the PTW EPID QC PHANTOM^®^.^11^

## Materials and methods

**FIGURE 1.**
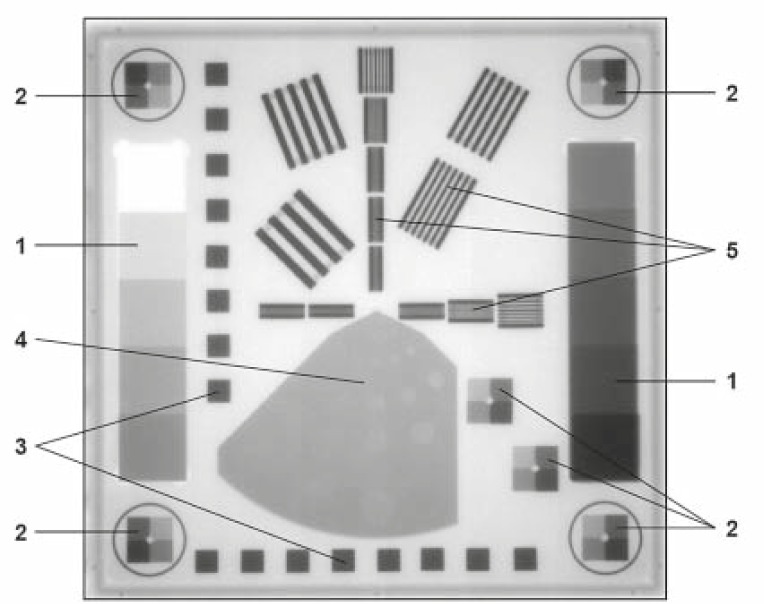
Structure of the PTW EPID QC PHANTOM^®^, 1. Signal linearity and signal noise ratio, 2. Isotropy of signal linearity, 3. Geometric isotropy (distortion), 4. Low-contrast resolution, 5. High-contrast resolution (MTF).

The PTW EPID QC PHANTOM^®^ was placed on the homogeneous part of the tabletop taking into account the divergence of the beam so that the whole phantom was in the image.^12^ The acquired images were analysed with the epidSoft^®^ 2.0 computer program.^13^ In our study we were interested not only in the quality of the images but also in the results given by the software for different file formats of the same image, such as JPEG, DCM, BMP, TIF, etc. We investigated the effect of different doses on the quality of the images. Figure 1 shows the phantom elements that were used for the calculation of different parameters.

*Linearity of Copper Steps Wedges:* two copper steps were used for linearity determination. The copper steps are designed in such a way that a range of 0% to 50% absorption rate is covered for a typical accelerator at 6 MV beam energy. The linearity curve was calculated from the mean of the gray values of each of the copper steps. The results were the levels 0%, 5%, 10%, 15%, 20%, 25%, 30%, 35%, 40% and 50%. For the display an additional 45% value was calculated from the 40% and 50% values by linear interpolation.

The *Local dependence of Linearity* was determined by means of the brass steps in the corners and at the bottom right side of the phantom (Figure 1.2). Each of these six sets of brass steps consists of four steps, which cover approximately 10%, 20% and 40% absorption rate at 6 MV. Linearity curve is calculated for each block from the mean of the gray values of the steps.

*Signal-to-Noise Ratio (SNR)* was also determined by means of the two copper steps (Figure 1.1). The SNR was calculated for each absorption level of the copper step.

*Modulation Transfer Function (MTF) and High Contrast Resolution:* the regions denoted by number 5 in the middle area of the phantom in Figure 1 were used for the determination of the MTF and the high contrast resolution (in horizontal and vertical direction). The mean of the gray values of the lamellae (maximal) and the mean of the gray values of the gaps (minima) were determined for each lamella block.

**FIGURE 2.**
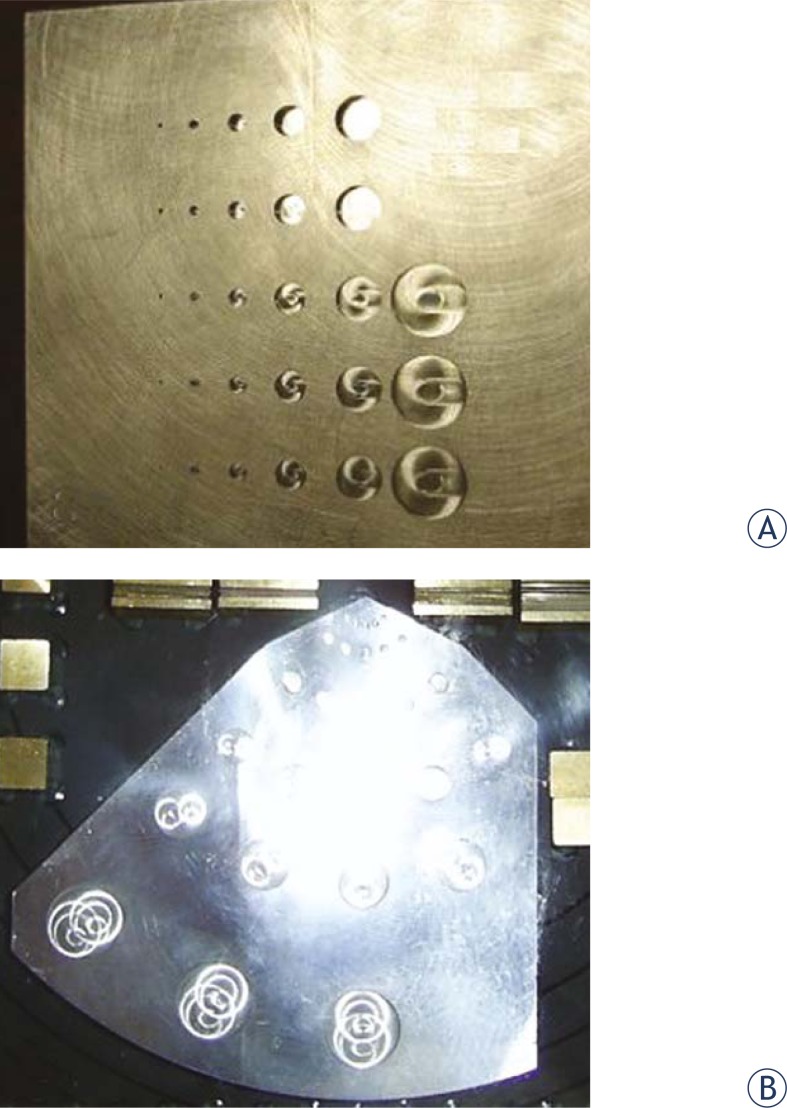
Controlling the low contrast resolution with (A) Las Vegas test tool and (B) PTW EPID QC PHANTOM^®^.

The *Low Contrast Resolution* was determined with the help of region 4 with 27 holes having different diameters and depths (Figure 1). For each hole the contrast difference of the hole and a specified area around the hole were calculated and represented in a column diagram. The diameters and depths of the holes are similar to those of the Las Vegas phantom, but Las Vegas phantom gives only visual information, while the PTW EPID QC PHANTOM^®^ also gives the numeric analysis (Figure 2).

In the case of Varian’s PortalVision^™^, the control software of the linac used 7 MUs for one portal image.^14^,^15^ The Siemens video based BeamView Plus^®^ was irradiated with 8, 10 and 16 MUs. The EPID was irradiated with 1, 2, 4, 6 and 8 MUs in the case of the Siemens OptiVue500aSi^®^ and the Elekta iView^®^.^16^–^18^ For portal films, we put both the phantom and the portal film cassette on the top of the treatment table. The Kodak Portal Localisation ReadyPack^®^ film was irradiated with 1, 2, 4, 6 and 10 MUs, while for Kodak X-OMAT V^®^ film we used 7, 20 and 40 MUs, because it’s lower sensitivity. We digitized the films with the LUMISYS Lumiscan^®^ 50 with two different softwares. One was the PTW’s Mephysto^®^ program, where we saved the images in TIF and PTW file format, and the other was the P2 System LumiDicom^®^ program^19^,^20^, where we saved the images in DCM and BMP file format. The reference values shall be determined during the acceptance test of the equipment. In the measurement protocol, the usable file format shall be defined since the implementation of DICOM is not complete at these systems.

## Results and discussion

**FIGURE 3.**
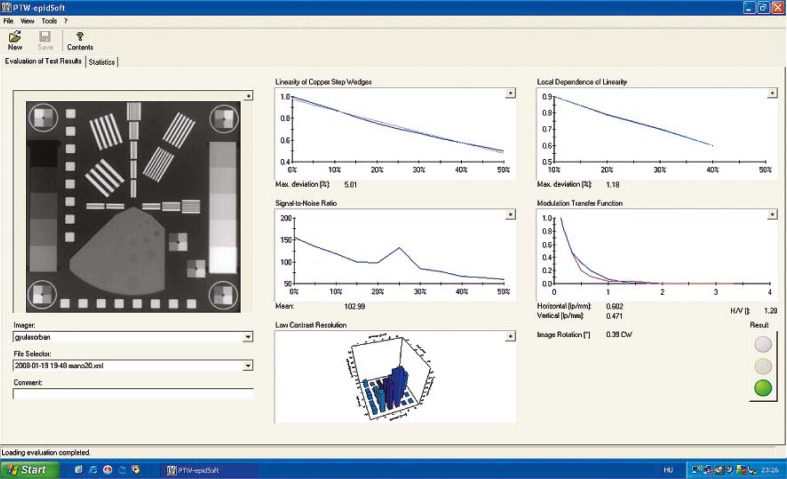
The image analysis with the epidSoft^®^2.0 program.

**FIGURE 5.**
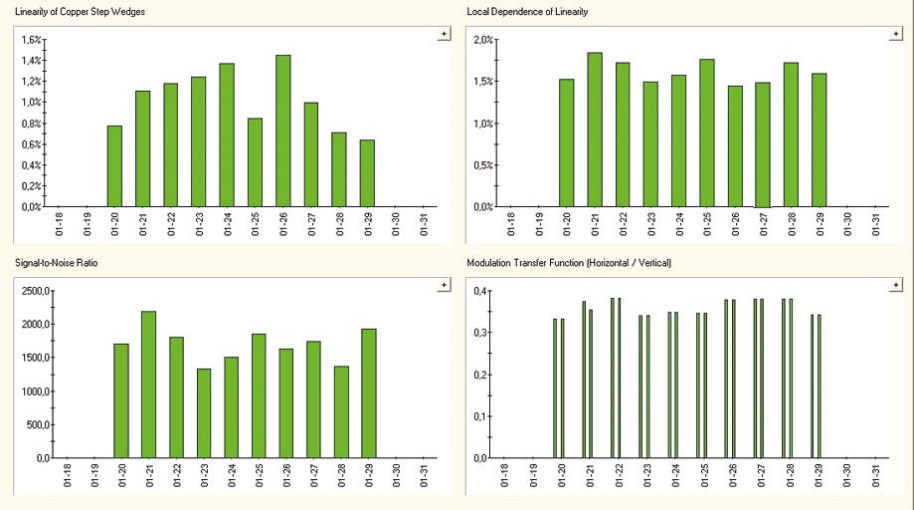
The graphic representation of the Varian PortalVision^™^ equipment’s stability test: A. linearity of copper step wedge, B. local dependence of linearity, C. MTF f(25) vertical and horizontal component, D. signal-to-noise ratio average value.

The epidSoft^®^2.0 program makes both, the numeric and the graphic analysis of the portal images; a screenshot can be seen in Figure 3. We analysed about 70 images taken under different conditions (Table 1). Comparing the f50 and f25 values of the MTF we resolved that the amorphous silicon EPID provides the best high contrast resolution. These results were very close to the MTF of the Kodak Portal Localisation ReadyPack^®^ film with the EC-L Lightweight^®^ cassette. For the MTF f50 and f30 we found few published data in the international literature 21–23; these are listed in Table 2. We also tested the constancy of the characteristics in the case of Varian’s PortalVision™ images with PTW EPID QC Phantom^®^. The graphic interpretation of the measurements is in Figure 5. In Figure 3, the upper left diagram, the measured values of daily Linearity of Copper Step Wedge curves are compared with those of calculated from the linear regression line of daily measurements on the base of equation 1.

[1.] $$\begin{array}{l} d_{\text{max}}=\text{max}\left[\frac{\left|y_{i}-I_{i}\right|}{I_{i}}\right]\\ i=0....N \end{array}$$

The linearity curve is given by the points (x_1_,y_1_), …(x_N_, y_N_) and the regression line is given by the points (x_1_,I_1_), …(x_N_, I_N_).

We have determined the 10 days stability of the system. The daily maxima are plotted vs. time in a 10 days interval in the upper left diagram of Figure 5. The maximum of the daily deviation is on an average 1.03 ± 0.27% representing a sufficient stability of the measurement system. A similar analysis was made for the Local Dependence of Linearity resulting in 1.62 ± 0.19% average daily maximum deviation. For the signal-to-noise curve, the average of mean value and standard deviation, were 1656 ± 189. The average value for MTF f50 was 0.247 ± 0.011 lp/mm and for MTF f25 we received 0.360 ± 0.018 lp/mm. Measurements shown in the Figure 5 represent a good stability of the system.

The signal-to-noise curve for the Siemens BeamView Plus^®^ and the Varian’s PortalVision^™^ depend of the image file format. In Figure 5 we can see the Siemens BeamView Plus^®^ SNR curves for DICOM, BMP and the inverse BMP file format. We supposed that two older generations of EPIDs had a DICOM implementation problem.^24^

**FIGURE 6.**
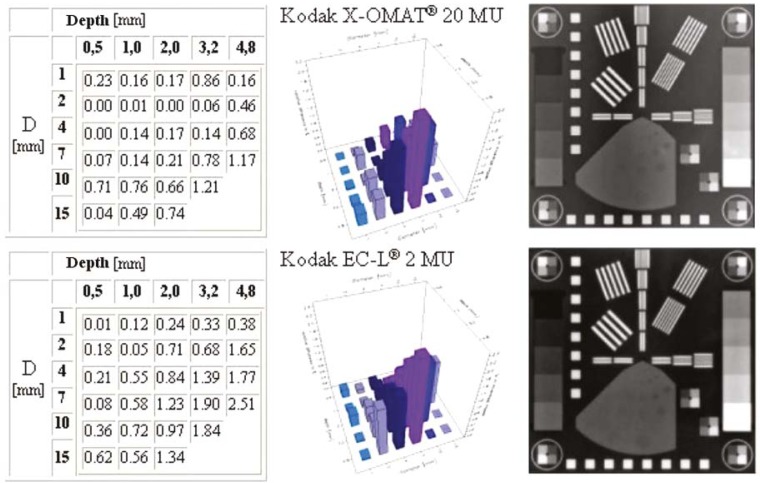
Comparison of results for low contrast resolution with PTW EPID QC PHANTOM^®^ for Kodak X-OMAT^®^ cassette with Kodak X-OMAT V^®^ film and the Kodak EC-L Lightweight^®^ cassette with Kodak Portal Localisation ReadyPack^®^ film.

If we use the Las Vegas phantom for quality control, then the image quality is acceptable when we can see 17 holes from 28 holes.^25^ We applied the same criteria for the PTW EPID QC PHANTOM^®^. All equipments gave good results, except the Kodak X-OMAT V^®^ film: we found only 9 holes after irradiating the film with a 10 times higher dose than the Kodak Portal Localisation ReadyPack^®^ film. The numeric and graphic interpretation of the low contrast resolution for portal films is in the Figure 6.

When the Kodak Portal Localisation ReadyPack^®^ film with EC-L Lightweight^®^ cassette was overexposed (Table 1), we received a too large value for Linearity of Copper Step Wedge and the image was unusable for the verification of patient setup.

Unfortunately, there is not a lot of published information regarding the physical characteristics of different EPIDs making it difficult to compare these results. On the base of physical characteristics, measured in this work, the authors suggest the use of aSi EPIDs producing the best image quality. Parameters of the EPIDs with scanning liquid ionisation chamber (SLIC) were very stable. The disadvantage of older versions of EPIDs like SLIC and VEPID is a pour DICOM implementation, and the modulation transfer function (MTF) values (f50 and f25) are less than that of aSi detectors.

## Figures and Tables

**FIGURE 4.**
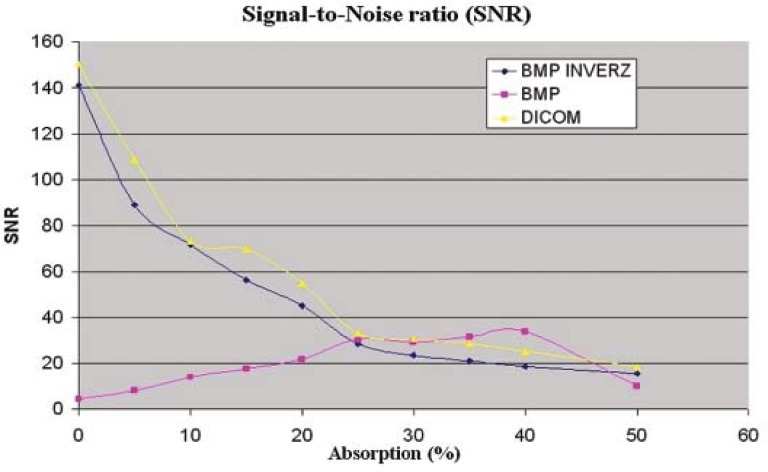
SNR as a function of absorption for Siemens BeamView Plus^®^ in the case of different file format: DICOM, bmp and inverse bmp.

**TABLE 1.** Results of the portal image analysis with the epidSoft^®^2.0 program for the different equipments

**Equipment**	**File format**	**MU**	**MFT**		**SNR**	**LCSW (%)**	**LDL (%)**
			**f50**	**f25**			
PortalVision^™^	dicom 3.0	7+7	0.288	0.402	52.8	0.57	1.43
PortalVision^™^	dicom RI	7+7	0.239	0.342	2532.7	0.67	1.63
PortalVision^™^	bmp	7+7	0.251	0.355	107.3	0.60	1.50
BeamView Plus^®^	dcm	8	0.307	0.437	38.1	12.9	3.24
BeamView Plus^®^ 15MV	bmp	8	0.225	0.378	40.7	11.0	2.16
BeamView Plus^®^	bmp	8	0.216	0.399	23.2	10.8	2.47
BeamView Plus^®^	bmp inverse	8	0.310	0.435	37.8	12.8	2.92
BeamView Plus^®^	bmp	10	0.242	0.402	54.2	11.6	2.75
BeamView Plus^®^	bmp	8+8	0.241	0.399	40.8	10.9	2.16
BeamView Plus^®^ 15MV	bmp	8+8	0.23	0.388	56.6	12.3	2.46
OptiVue500aSi^®^	dcm	1	0.317	0.574	93.3	6.33	2.45
OptiVue500aSi^®^	dcm	2	0.315	0.573	105.3	6.15	2.48
OptiVue500aSi^®^	dcm	4	0.315	0.569	95.3	6.16	2.37
OptiVue500aSi^®^	dcm	6	0.315	0.563	86.4	6.10	2.32
OptiVue500aSi^®^	dcm	8	0.315	0.563	72.9	6.08	2.23
Elekta iView^®^	bmp	1	0.323	0.597	115.3	5.07	1.58
Elekta iView^®^	bmp	2	0.324	0.602	102.9	5.01	1.18
Elekta iView^®^	bmp	4	0.321	0.576	99.2	5.06	1.28
Elekta iView^®^	bmp	6	0.315	0.572	90.8	5.03	1.48
Elekta iView^®^	bmp	8	0.305	0.539	72.4	4.67	1.34
X-OMAT^®^ film	bmp	20	0.322	0.609	248.1	4.62	1.52
X-OMAT^®^ film	tif	20	0.333	0.548	167.8	3.61	2.59
X-OMAT^®^ film	dicom RI	20	0.207	0.396	146.8	3.59	2.32
X-OMAT^®^ film	dicom RI	40	0.275	0.692	105.6	2.34	2.68
EC-L^®^ film	dicom RI	1	0.336	0.596	90.2	5.01	1.89
EC-L^®^ film	dicom RI	2	0.316	0.569	100.7	4.71	2.36
EC-L^®^ film	dicom RI	4	0.331	0.574	92.5	5.00	1.71
EC-L^®^ film	dicom RI	6	0.306	0.563	88.6	6.84	3.96
EC-L^®^ film	tif	1	0.324	0.572	113.1	4.97	1.89
EC-L^®^ film	tif	2	0.312	0.563	119.9	4.66	2.48
EC-L^®^ film	tif	4	0.324	0.584	110.2	4.96	1.71
EC-L^®^ film	tif	10	0.291	0.600	88.2	10.02	3.63

** LCSW, Linearity of Copper Step Wedge

*** LDL, Local Dependence of Linearity

**TABLE 2.** Demonstration of the quantities to be used for the quality control of the EPIDs found in the references

**EPID**	**Pixel matrix**	**Pixel size (mm)**	**Dose (MU)**	**CNR**	**f50 (lp/mm)**	**f30 (lp/mm)**
Clements at al. 2002, PIPSpro® QC-3V fantom [118]						
Varian aS500™	512 × 384	0.78	5	260	0.392	0.600
Elekta iViewGT®	1024 × 1024	0.4	100	448	0.461	0.767
Siemens FP-A	1024 × 1024	0.4	100	611	0.454	0.696
Hermann at al. 2001. [119]						
BeamView Plus®	512 × 512				0.204	
PortalVision™	256 × 256				0.258	
Wong, 1999. [120]						
BeamView Plus®	512 × 512				0,214	
					0,192 (15 MV)	
PortalVision™	256 × 256				0,258	
